# Improved effect of a mitochondria-targeted antioxidant on hydrogen peroxide-induced oxidative stress in human retinal pigment epithelium cells

**DOI:** 10.1186/s40360-020-00471-w

**Published:** 2021-01-20

**Authors:** Myung Hee Kim, Do-Hun Kim, Su Geun Yang, Dae Yu Kim

**Affiliations:** 1 Inha Research Institute for Aerospace Medicine, Inha University, Incheon, 22212 South Korea; 2 Department of Biomedical Science, BK21 FOUR Program in Biomedical Science & Engineering, Inha University College of Medicine, Incheon, 22332 South Korea; 3 Department of Electrical Engineering, College of Engineering, Inha University, Incheon, 22212 South Korea; 4 Center for Sensor Systems, Inha University, Incheon, 22212 South Korea

**Keywords:** Age-related macular degeneration, Retinal pigment epithelium, Mitochondrial function, Antioxidants

## Abstract

**Background:**

Oxidative damage to retinal pigment epithelial (RPE) cells contributes to the development of age-related macular degeneration, which is among the leading causes of visual loss in elderly people. In the present study, we evaluated the protective role of triphenylphosphonium (TPP)-Niacin against hydrogen peroxide (H_2_O_2_)-induced oxidative stress in RPE cells.

**Methods:**

The cellular viability, lactate dehydrogenase release, reactive oxygen species (ROS) generation, and mitochondrial function of retinal ARPE-19 cells were determined under treatment with H_2_O_2_ or pre-treatment with TPP-Niacin. The expression level of mitochondrial related genes and some transcription factors were assessed using real-time polymerase chain reaction (RT-qPCR).

**Results:**

TPP-Niacin significantly improved cell viability, reduced ROS generation, and increased the antioxidant enzymes in H_2_O_2_-treated ARPE-19 cells. Mitochondrial dysfunction from the H_2_O_2_-induced oxidative stress was also considerably diminished by TPP-Niacin treatment, along with reduction of the mitochondrial membrane potential (MMP) and upregulation of the mitochondrial-associated gene. In addition, TPP-Niacin markedly enhanced the expression of transcription factors (PGC-1α and NRF2) and antioxidant-associated genes (especially HO-1 and NQO-1).

**Conclusion:**

We verified the protective effect of TPP-Niacin against H_2_O_2_-induced oxidative stress in RPE cells. TPP-Niacin is believed to protect against mitochondrial dysfunction by upregulating antioxidant-related genes, such as PGC-1α, NRF2, HO-1, and NQO-1, in RPE cells.

**Supplementary Information:**

The online version contains supplementary material available at 10.1186/s40360-020-00471-w.

## Background

Age-related macular degeneration (AMD) is one of the most common causes of irreversible blindness in the elderly population in developed countries. There are two major forms of AMD: non-neovascular dry form of AMD affecting approximately 85–90% of patients and neovascular exudative wet form affecting the remaining 10–15% of patients [1, 2]. Dry AMD (atrophy AMD) is caused by changes in the pigmentation of the retinal pigment epithelial (RPE) cells and subretinal deposits owing to lipid and protein accumulation between these cells and Bruch’s membrane, a condition termed as drusen. These processes finally result in RPE cell death, photoreceptor dysfunction, and loss of vision [1–3]. At present, anti-vascular endothelial growth factor (anti-VEGF) therapy has enabled extraordinary improvements in wet AMD; however, effective treatments for dry AMD are not yet available [4, 5].

Despite being a condition of unknown etiology, oxidative stress is considered as a major influence on RPE cells in AMD pathophysiology [5–7]. RPE cells have high metabolic rates with enriched mitochondrial population, and the oxidative phosphorylation process produces adenosine triphosphate (ATP), indicating the generation of high amounts of reactive oxygen species (ROS) [8]. Thus, RPE cells exist in an environment of abundant endogenous ROS, and the long-term accumulation of such oxidative damage causes dysfunctions in the RPE cells and increases their susceptibility to oxidative stress. The ROS predominantly target mitochondria and destroy their membrane integrity, dissipating the mitochondrial membrane potential (ΔΨm, MMP), causing mitochondrial dysfunction, and governing cell survival. Indeed, intramitochondrial oxidative stress is connected with processes ruling cell survival, such as mitochondrial flexibility, apoptosis, and autophagy, in AMD [9]. Therefore, protecting RPE cells from oxidative damage is necessary for preventing or weakening AMD.

**Fig. 1:**
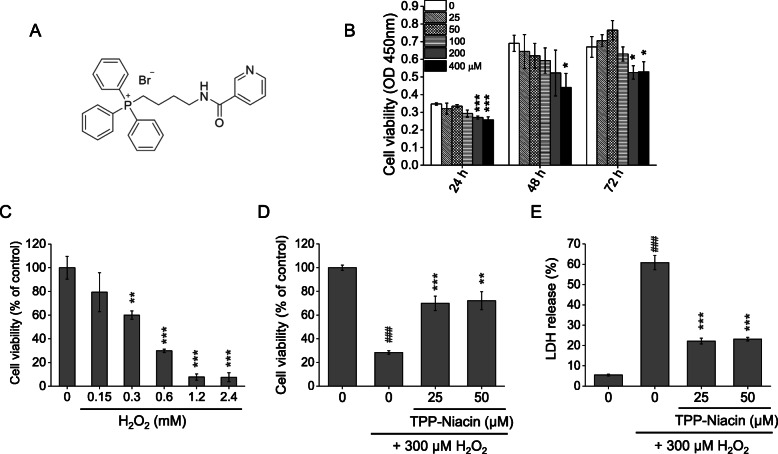
Protective effects of TPP-Niacin against H_2_O_2_-induced cytotoxicity in ARPE-19 cells. **a** Chemical structure of TPP-Niacin. **b** RPE cells were treated with TPP-Niacin (25–400 μM) or 0.1% DMSO (vehicle control) for 24–72 h and the cell viabilities were measured using the CCK-8 assay. **c** Cells were treated with H_2_O_2_ (0.15–2.4 mM) for 24 h and cell viabilities were measured. Cells were pretreated with TPP-Niacin at indicated concentrations or 0.1% DMSO (vehicle control) for 2 h and then incubated with or without 300 μM H_2_O_2_ for a further 24 h. Cell viabilities and LDH release were measured by the CCK-8 assay (**d**) and LDH assay (**e**), respectively. ### *P* < 0.001 versus control group and **P < 0.01, ***P < 0.001 versus the H_2_O_2_-treated group were considered statistically significant differences

To improve the therapeutic effects and diminish the side effects of chemicals, researchers have investigated strategies for subcellular targeting, especially the mitochondria. The selective targeting of antioxidants toward mitochondria by covalent conjugation with the lipophilic triphenylphosphonium (TPP) cation is a popular method [10–12]. TPP, which is a well-known mitochondrial targeting moiety, is a membrane-permeant lipophilic cation that is quickly accumulated several fold within the mitochondria in vivo and is controlled by the large MMP [13]. Since the development of mitochondrial target compounds, there have been several reported mitochondria-targeting antioxidants, including MitoQ [14], MitoC [15], MitoE [16], and TPP-IOA [17]. Most of these studies employed traditional antioxidants such as vitamin C, vitamin E, and oleic acid to obtain TPP conjugates. Among these vitamins, B3 (niacin or nicotinic acid) is widely recognized as a potent antioxidant that also exerts powerful lipid-lowering effects at high concentrations [18–21]. However, based on a literature search, neither the synthesis of TPP-conjugated niacin (TPP-Niacin, Fig. 1a) for mitochondrial targeting nor its antioxidant effects have been demonstrated. Therefore, in this study, we synthesized TPP-conjugated niacin (TPP-Niacin) and investigated its protective effects on RPE cells for hydrogen peroxide (H_2_O_2_)-induced damage. We also evaluated the molecular actions underlying the effects of TPP-Niacin on H_2_O_2_-stimulated ARPE-19. In brief, we confirmed that TPP-Niacin exerted a protective role against H_2_O_2_-induced cytotoxicity and mitochondrial dysfunction via upregulation of the antioxidant-associated genes in RPE cells.

## Methods

The human ARPE-19 cell line was purchased from American Type Culture Collection (ATCC, Manassas, VA, USA). Dulbecco’s modified Eagle’s medium: nutrient mixture F-12 media (DMEM/F12), fetal bovine serum (FBS), penicillin/streptomycin, and 2,7-dichlorofluorescein diacetate (H_2_DCF-DA) were purchased from Thermo Fisher Scientific (Wilmington, DE, USA). Hydrogen peroxide, dihydroethidium (DHE), JC-10 assay kit, N-acetyl-cysteine (NAC), and carbonyl cyanide m-chlorophenyl hydrazone (CCCP) were purchased from Sigma-Aldrich (St. Louis, MO, USA, owned by Merck KGA). TPP-Niacin was chemically synthesized according to a previous report and patent application, and niacin was used as the reference control [12, 14, 22, 23]. The Cell Counting Kit-8 (CCK-8) and lactate dehydrogenase (LDH) assays were purchased from Dojindo Molecular Technologies (Japan). The kits used to determine the superoxide dismutase 1 and 2 (SOD1 and SOD2), catalase and glutathione peroxidase activities, and tetramethylrhodamine ethyl ester (TMRE) reagent were obtained from Abcam (UK).

### Cell culture

The human ARPE-19 cells were routinely maintained in DMEM/F12 supplemented with 10% FBS and 1% penicillin/streptomycin. The cells were incubated at 37 °C under atmospheric conditions at 5% CO_2._

### Cell viability assay

The ARPE-19 cells were placed in 96-well plates with 5× 10^3^ cells/well and incubated for 24 h. The next day, the cells were exposed to different concentrations of TPP-Niacin for 24, 48, and 72 h. The cell viabilities were then assessed using the CCK-8 assay. In brief, 10 μL of the CCK-8 reagent was added to each sample and incubated for 1 h. Then, the absorbance values were measured at 450 nm using a microplate reader (Bio-Tek, Winooski, VT, USA). To evaluate suitable H_2_O_2_ concentrations for oxidative damage and cytotoxic induction, the cells were seeded in 96-well plates for 24 h and incubated with various concentrations of H_2_O_2_ for another 24 h. Then, the cell viabilities were evaluated by CCK-8 using the same method as before. To examine the protective effects of TPP-Niacin against H_2_O_2_-induced oxidative damage, the seeded cells were pretreated with different concentrations of TPP-Niacin for 2 h, followed by adding to 300 μM H_2_O_2_ in TPP-Niacin contained media for an additional 24 h. Then, the cell viabilities were evaluated using CCK-8. All samples were prepared in triplicate, and each experiment was repeated three times.

### Lactate dehydrogenase (LDH) release assay

Cell cytotoxicities were determined by the LDH released from damaged cells using the cytotoxicity LDH assay kit. In brief, ARPE-19 cells were placed in 96-well plates (5 × 10^3^ cells/well) and pretreated with different concentrations of TPP-Niacin for 2 h, followed by adding to 300 μM H_2_O_2_ in TPP-Niacin contained media for 24 h. The background of LDH in the growth medium was measured and subtracted from all test samples. The basal levels of LDH (0% cell death), as measured in the supernatants of the vehicle-treated cells, and maximal levels of LDH (100% cell death) measured by lysis buffer induction for complete cell death, were averaged and used to calculate the percentage of cell death as per the manufacturer’s protocol.

### Measurement of ROS

The generation of intracellular ROS was examined using the ROS detection reagent according to manufacturer instructions. Briefly, cells were grown in a 96-well black plate (Eppendorf Ltd., Germany) and subjected to different treatments with/without TPP-Niacin and H_2_O_2._ Then, the cells were incubated with 5 μM H_2_DCF-DA or 10 μM DHE at 37 °C for 20 min. The fluorescence intensities were measured using the fluorescence plate reader (Bio-Tek) at Ex./Em. = 495/527 nm for H_2_DCF-DA and Ex./Em. = 535/610 nm for DHE. Then, the H_2_DCF-DA-stained cell images were obtained with a fluorescent microscope IX51 with DP controller (Olympus Optical, Japan). All samples were prepared in triplicate, and each experiment was repeated three times.

### Antioxidant enzyme activity

The ARPE-19 cells (5 × 10^6^ cells/well) were plated onto 100 mm cell culture dishes and pretreated with different concentrations of TPP-Niacin for 2 h, followed by adding to 300 μM H_2_O_2_ in TPP-Niacin contained media for 24 h; then, the cells were collected in clean tubes with 100 μL Pro-Prep (Intron, Korea) lysis buffer for 20 min on ice after washing with phosphate buffered saline (PBS). Next, the supernatant was carefully collected after centrifugation and protein concentration was calculated with a bicinchoninic acid protein assay kit (Thermo Fisher Scientific). The intracellular activities of SOD1, SOD2, CAT, and GPx were detected with commercial kits (Abcam, UK) according to manufacturer directions, and the results were shown as percentages of the untreated groups.

### MMP and staining

The MMP assay was conducted as per manufacturer instructions for the JC-10 MMP assay kit. In brief, ARPE-19 cells (5 × 10^3^ cells/well) seeded in the 96-well clear bottom black plates were pretreated with different concentrations of TPP-Niacin for 2 h, followed by adding to 300 μM H_2_O_2_ in TPP-Niacin contained media. After 24 h, the JC-10 assay solution was added to the cells, and the plate was incubated in the dark for 30 min. Following incubation, the assay buffer B was added, and the fluorescent intensities were measured at 490/525 nm and 540/590 nm using a multimode plate reader (Bio-Tek). The ratio of red/green fluorescent intensity was used to determine the MMP. CCCP and NAC were used as the positive and antioxidant control, respectively. All samples were examined in triplicate, and each experiment was repeated three times. To visualize the effects of TPP-Niacin on the MMP, cells were stained with TMRE reagent and analyzed using an inverted fluorescence microscope IX51 with DP controller (Olympus Corporation, Japan) at Ex./Em. = 549/575 nm.

### Transmission electron microscopy

The treated ARPE-19 cells were washed three times with 0.1 M PBS and fixed overnight in 3% glutaraldehyde at 4 °C and 1% osmium tetroxide solution as additional fixation for 30 min. The cells were dehydrated with a graded series of ethanols and then embedded in epoxy resin. The embedded sample was cut into ultrathin sections around 60 nm using an ultramicrotome (RMC MT-XL; RMC Products, Tucson, AZ, USA) and observed with a transmission electron microscope (Hitachi H-7100, Japan).

### RNA collection and quantitative PCR

The total RNA was collected using Trizol reagent (Thermo Fisher Scientific) and resuspended in RNAse-free water. The concentration of each sample was then determined using the NanoDrop 1000 spectrophotometer (Thermo Fisher Scientific). Reverse transcription was subsequently performed with 1 μg of RNA to produce the complimentary DNA (cDNA) using the SensiFast cDNA synthesis kit (Bioline, London, UK). To measure the gene expression, a quantitative polymerase chain reaction (qPCR) was performed using 3 μL of the cDNA template and the Power SYBR Green Master Mix (Thermo Fisher Scientific) on a StepOnePlus apparatus (Applied Systems, USA). All samples were examined in triplicate, and each experiment was repeated three times. Data were normalized to the mean expression of the housekeeping gene using GAPDH and quantified using the 2−^ΔΔCT^ method. The primer sequences used are summarized in Table 1.

**Table 1:** Primer sequences used in this study

Target gene	Forward sequence (5′-3′)	Reverse sequence (5′-3′)
ATP5O	CGCTATGCCACAGCTCTTTA	AAGGCAGAAACGACTCCTTG
COX4I1	GGCATTGAAGGAGAAGGAGA	TCATGTCCAGCATCCTCTTG
COX5B	GAGGTGGTGTTCCCACTGAT	CAGACGACGCTGGTATTGTC
NDUFB5	CTTCCTCACTCGTGGCTTTC	TCTGGGACATAGCCTTCTGG
FIS1	GACATCCGTAAAGGCATCGT	ACAGCAAGTCCGATGAGTCC
MFN1	TGCCCTCTTGAGAGATGACC	TCTTTCCATGTGCTGTCTGC
MFN2	ATGCATCCCCACTTAAGCAC	GCAGAACTTTGTCCCAGAGC
TFAM	TAAGACTGCAAGCAGCGAAG	TTCTCAGTTTCCCAGGTGCT
POLG	TGCAGTGAGGAGGAGGAGTT	CCCAGGTAAGTGCCATGAGT
SOD1	GAAGGTGTGGGGAAGCATTA	CTTTGCCCAAGTCATCTGCT
SOD2	AAACCTCAGCCCTAACGGTG	GCCTGTTGTTCCTTGCAGTG
CAT	GATAGCCTTCGACCCAAGCA	AGAAGGCTGTTGTTCCGGAG
GPX1	AGTCGGTGTATGCCTTCTCG	CAAACTGGTTGCACGGGAAG
TXN2	TGGTGGCCTGACTGTAACAC	CACCGCTGACACCTCATACT
PRDX3	TGCATGTGGAAGAACGAGCT	TCCACTGAGACTGCGACAAC
PRDX5	AGGGTGTGCTGTTTGGAGTT	TCCACATTCAGGGCCTTCAC
PRDX6	CAGCTCGTGTGGTGTTTGTT	AGATGGGAGCTCTTTGGTGA
HMOX1	AGTCTTCGCCCCTGTCTACT	GCTTGAACTTGGTGGCACTG
NQO1	AAAGGACCCTTCCGGAGTAA	CGTTTCTTCCATCCTTCCAG
PGC1a	CAAGCAAAGGGAGAGGCAGA	ACCTGCGCAAAGTGTATCCA
PGC1b	TGGGCTGAGTTCTCCATTCT	TGAAGCTGCGATCCTTACCT
ESRRA	TCGCTGTCTGACCAGATGTC	AGGGCCAAGGCCTTTAGTAG
FOXO1	GCATCCATGGACAACAACAG	AGATGGCGGGTACACCATAG
FOXO3	CATCATGGCAAGCACAGAGT	GAGCGTGATGTTATCCAGCA
NRF1	CTTACAAGGTGGGGGACAGA	CAATGTCACCACCTCCACAG
NRF2	GCGACGGAAAGAGTATGAGC	ACGTAGCCGAAGAAACCTCA
PPARA	CCCTTTTTGTGGCTGCTATC	ATCCGACTCCGTCTTCTTGA
SIRT1	CCATGGCGCTGAGGTATATT	TCTCCATCAGTCCCAAATCC
GAPDH	ACCCAGAAGACTGTGGATGG	TTCTAGACGGCAGGTCAGGT

### Statistics

Statistical analyses were performed using the GraphPad Prism 5 software (GraphPad Software Inc., La Jolla, CA, USA), and all data were presented as mean±SD. The Student’s t-test was used to calculate statistical significance between paired groups. In this study, the statistical significances are denoted as follows: ns P > 0.05, ∗*P* < 0.05, ∗∗*P* < 0.01, and ∗∗∗*P* < 0.001.

## Results

### Cell viability and protective effect of TPP-niacin on ARPE-19 cells

To evaluate the optimal concentration of TPP-Niacin (Fig. 1a) that can be used without causing cytotoxicity, the ARPE-19 cells were incubated with various concentrations of TPP-Niacin for 24, 48, and 72 h. As shown in Fig. 1b, the TPP-Niacin at 25 and 50 μM concentrations did not show any cytotoxicities in the ARPE-19 cells compared to the control group, whereas concentrations between 100 and 400 μM attenuated the cell viabilities at 48 and 72 h. Thus, 25 and 50 μM of TPP-Niacin were used in the subsequent experiments. To determine a suitable H_2_O_2_ concentration for oxidative damage assessment, the cells were exposed to various concentrations of H_2_O_2_ for 24 h. The H_2_O_2_ treatment (around 300 μM) significantly reduced cell viability, resulting in 46.3% cell death (Fig. 1c). Therefore, the H_2_O_2_ concentration of 300 μM was utilized in the subsequent experiments. To test the protective effects of TPP-Niacin on H_2_O_2_-induced cell death, the cells were treated with TPP-Niacin for 2 h before being exposed to H_2_O_2_ for 24 h. As shown in Fig. 1d, the pretreatments with 25 and 50 μM of TPP-Niacin significantly improved H_2_O_2_-induced reductions of ARPE-19 cells (at 25 μM: 70 ± 6.1%; at 50 μM: 72 ± 7.5%). The protective effects of TPP-Niacin were also assessed by the LDH assay. The TPP-Niacin pre-treated cells for 2 h significantly reduced H_2_O_2_-induced LDH levels (Fig. 1e). In addition, to compare the protective effects of niacin and TPP-Niacin, the cell viability assays were performed with and without oxidative stress. The test compounds did not show any cytotoxicities, and the pretreatment of cells with niacin, TPP-Niacin, and NAC for 2 h significantly protected the cells from H_2_O_2_-induced cell death (Supplementary Figure 1a and 1b).

### TPP-niacin suppressed H_2_O_2_-induced oxidative stress in ARPE-19 cells

**Fig. 2:**
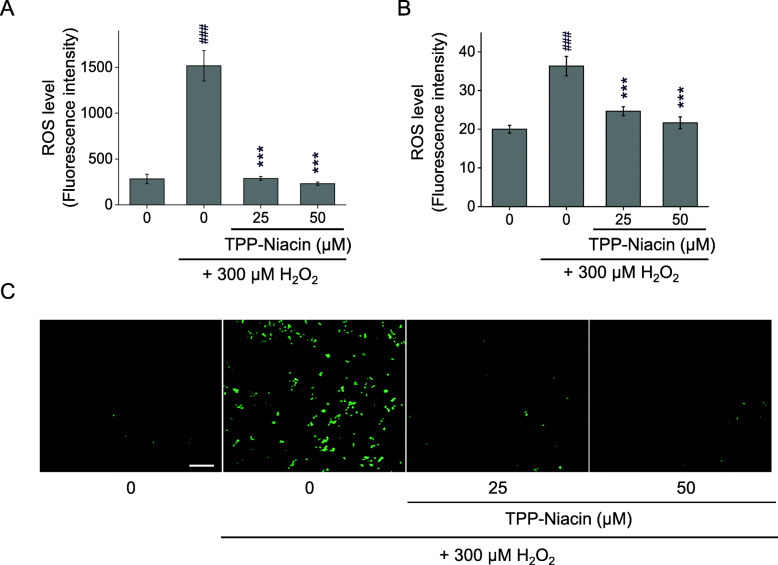
Protective effects of TPP-Niacin against H_2_O_2_-induced ROS generation in ARPE-19 cells. The cells were pretreated with TPP-Niacin at 25 and 50 μM or 0.1% DMSO (vehicle control) for 2 h and then incubated with or without 300 μM H_2_O_2_ for a further 24 h, and ROS generation was measured by the H_2_DCF-DA (**a**) and DHE (**b**). Representative cell images were assessed by H_2_DCF-DA staining (**c**). ### *P* < 0.001 versus control group and ***P < 0.001 versus the H_2_O_2_-treated group were considered statistically significant differences. The scale bar in (**c**) represents 100 μm. H_2_DCF-DA: dichlorofluorescein diacetate, DHE: dihydroethidium

**Fig. 3:**
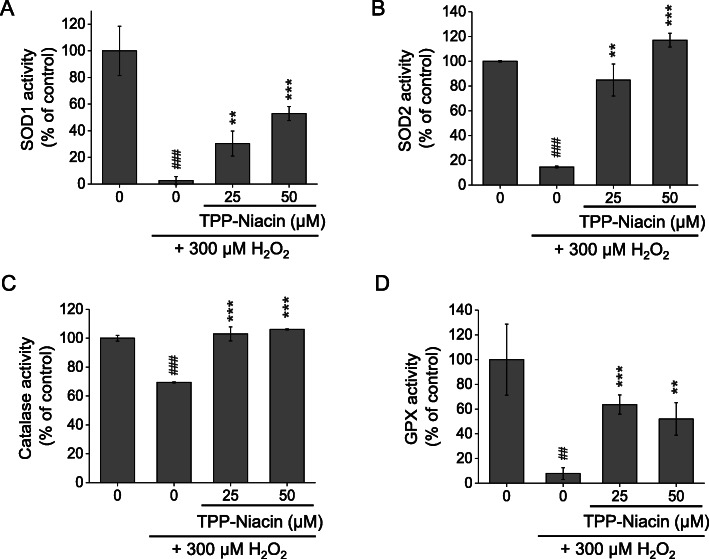
TPP-Niacin improved H_2_O_2_-induced decreasing antioxidant status in ARPE-19 cells, which were pre-treated with TPP-Niacin at 25 and 50 μM for 2 h and then treated with H_2_O_2_ (300 μM) for 24 h. The levels of SOD1 (**a**), SOD2 (**b**) Catalase, (**c**) and GPx activity (**d**) were measured to assess the levels of antioxidant activities. ## *P* < 0.01, ### *P* < 0.001 versus control group and **P < 0.01, ***P < 0.001 versus the H_2_O_2_-treated group were considered statistically significant differences. SOD1 and 2: superoxide dismutase 1 and 2, and GPx: glutathione peroxidase

The excessive accumulation of ROS is regarded as one of the main sources of cell damage induced by H_2_O_2_. Intracellular ROS signals were quantified using a fluorescence probe, H_2_DCF-DA and DHE reagent, in the ARPE-19 cells. As shown in Fig. 2, compared with the control group, 300 μM H_2_O_2_ caused a significant increase in the fluorescent intensity of H_2_DCF-DA (a) and DHE (b) in the ARPE-19 cells. However, pretreatment with TPP-Niacin in the ARPE-19 cells markedly decreased the ROS levels compared to H_2_O_2_ treatment alone (Fig. 2a and b). The suppressive activity of TPP-Niacin was also observed in the fluorescence image (H_2_DCF-DA), as illustrated in Fig. 2c. Meanwhile, to determine the role of the antioxidant enzymes for protective effects by TPP-Niacin against oxidative damage, the expressions of major antioxidant enzymes, including SOD1, SOD2, catalase (CAT), and GPx were measured by ELISA. The pretreatment with TPP-Niacin effectively replenished the activities of SOD1 and SOD2 in the ARPE-19 cells, which were earlier decreased by treatment with 300 μM H_2_O_2_ (Fig. 3a and b). Additionally, the CAT activity was significantly improved by pretreatment with TPP-Niacin compared to treatment with H_2_O_2_ alone (Fig. 3c). As shown in Fig. 3d, pretreatment with TPP-Niacin markedly enhanced the GPx level, which was almost abrogated by treatment with H_2_O_2_. In addition, the ARPE-19 cells pretreated with niacin and TPP-Niacin showed marked reductions in H_2_O_2_-induced ROS production. As expected, TPP-Niacin exerted a somewhat higher protective effect against oxidative stress compared to niacin-treated cells (as shown in Supplementary Figure 2A and 2B).

### TPP-niacin decreased H_2_O_2_-induced change of MMP and mitochondrial morphology

**Fig. 4:**
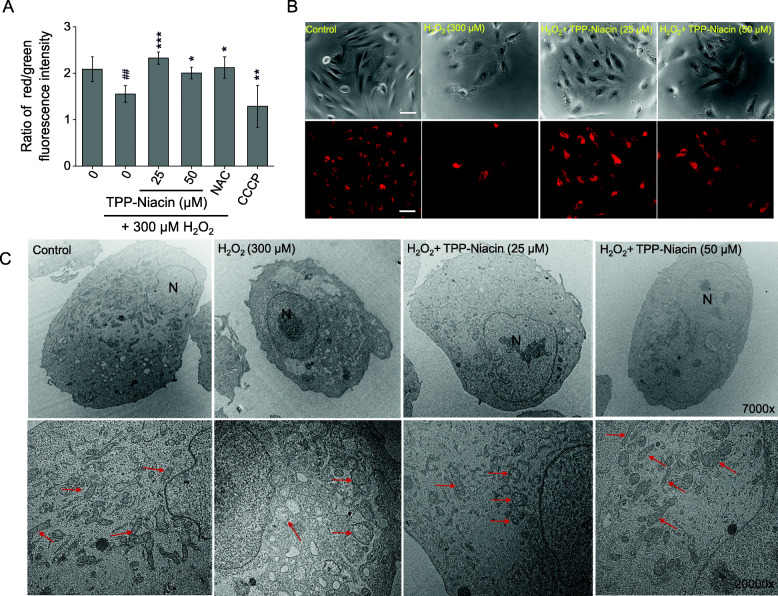
TPP-Niacin attenuated H_2_O_2_-induced mitochondrial membrane potential (△Ψm) losses and mitochondrial morphologies of ARPE-19 cells. After pretreatment with 25 and 50 μM TPP-Niacin for 2 h, ARPE-19 cells were incubated with or without 300 μM H_2_O_2_ for another 24 h. Quantification of red/green fluorescence intensities was determined by the JC-10 assay (**a**) and △Ψm was determined by TMRE staining. **b** CCCP (40 μM) and NAC (4 mM) were used as the positive and antioxidant controls, respectively. **c** Representative electron microscope images of the mitochondrial shape in the ARPE-19 cells treated without H_2_O_2_, with 300 μM H_2_O_2_ alone or with 300 μM H_2_O_2_ and 25 or 50 μM TPP-Niacin for 24 h. ## *P* < 0.01 versus control group and *P < 0.05, **P < 0.01, ***P < 0.001 versus the H_2_O_2_-treated group were considered statistically significantly different. Scale bar represents 50 μm. TMRE: tetramethylrhodamine ethyl ester, CCCP: carbonyl cyanide m-chlorophenyl hydrazine, NAC: N-acetyl-cysteine

Mitochondrial dysfunction causes loss of MMP (△Ψm). To determine whether TPP-Niacin could decrease H_2_O_2_-induced △Ψm loss, the △Ψm of the ARPE-19 cells was evaluated by analyzing the red/green fluorescence intensity ratio via the JC-10 assay. As shown in Fig. 4a, the ARPE-19 cells were exposed to 300 μM H_2_O_2_ and resulted in decrease of red/green fluorescence intensity ratio, indicating △Ψm dissipation, similar to CCCP, which is a mitochondrial oxidative phosphorylation uncoupler. However, pretreatment with TPP-Niacin at 25 and 50 μM for 2 h improved the H_2_O_2_-induced △Ψm loss to the same extent as the antioxidant positive control (NAC), as shown in Fig. 4a. The same tendency was observed in ARPE-19 cells pretreated with TPP-Niacin, when compared with those treated with H_2_O_2_ alone, using the TMRE reagent for visual illumination of the mitochondria (Fig. 4b). Interestingly, pretreatment with the parent compound (niacin) for 2 h did not show any improvement in the H_2_O_2_-induced △Ψm loss (Supplementary Figure 3); thus, the results reveal that TPP-Niacin could successfully be used as a target for mitochondria. Additionally, the mitochondrial morphology was characterized by electron microscopy (EM) (Fig. 4c). The H_2_O_2_-treated cells showed less dense cytoplasms and larger numbers of swollen mitochondria with disrupted cristae, whereas these appeared rather uniform, including intact cristae of the mitochondria morphology, in the control group. Although some mitochondria presented a disorganized structure with disturbed cristae and swollen appearance in the TPP-Niacin pretreated cells, pretreatment with TPP-Niacin revealed diminished mitochondrial damage in the ARPE-19 cells (Fig. 4c).

### TPP-niacin increased expression of OXPHOS and mitochondrial related genes

**Fig. 5:**
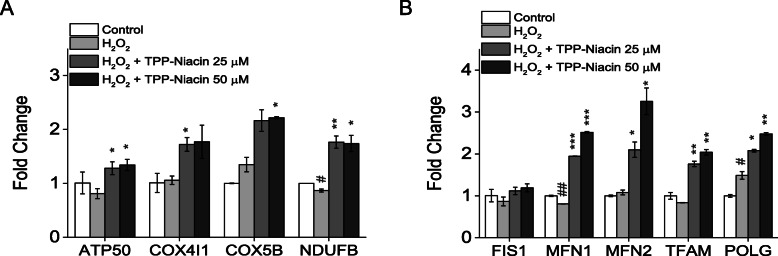
TPP-Niacin upregulated expression of OXPHOS target genes and mitochondrial dynamics genes in ARPE-19 cells. Relative expression of OXPHOS genes (**a**) and mitochondrial dynamic genes (FIS1, MFN1, and MFN2) and replication genes (TFAM and POLG) (**b**) in ARPE-19 treated for 2 h with 25 or 50 μM TPP-Niacin and then incubated with or without 300 μM H_2_O_2_ for a further 24 h. # *P* < 0.05, ## *P* < 0.01 versus control group and *P < 0.05, **P < 0.01, ***P < 0.001 versus the H_2_O_2_-treated group were considered statistically significantly different

To understand the molecular mechanisms of the protective effects of TPP-Niacin on mitochondrial biogenesis, the expression of mitochondrial respiration and mitochondrial dynamics genes were studied by real-time quantitative PCR (RT-qPCR). As expected, the expressions of OXPHOS component genes, ATP synthase subunit O (ATP5O), COX4I1 (cytochrome c oxidase subunit 4 isoform 1), cytochrome c oxidase subunit 5B (COX5b), and NADH dehydrogenase (ubiquinone) 1 beta subcomplex, 5 (NDUFB5), were significantly increased by pretreatment with TPP-Niacin in the ARPE-19 cells, as compared to H_2_O_2_ treatment alone and the control group (Fig. 5a). As illustrated in Fig. 5b, the mRNA expression levels of mitochondrial dynamics genes (fission 1 (FIS1), mitofusin 1 and 2 (MFN1 and 2)), mitochondrial DNA replication (polymerase (DNA directed) gamma (POLG)), and transcription gene (transcription factor a, mitochondrial (TFAM)), were significantly elevated by the TPP-Niacin compared to the H_2_O_2_ treated group. These results suggest that the upregulation of mitochondrial biogenesis genes intermediating the protective effect of TPP-Niacin on H_2_O_2_-induced cell damage in ARPE-19 cells.

### TPP-niacin increased expression of mitochondrial biogenesis related genes via upregulated PGC-1a/NRF2 Axis

**Fig. 6:**
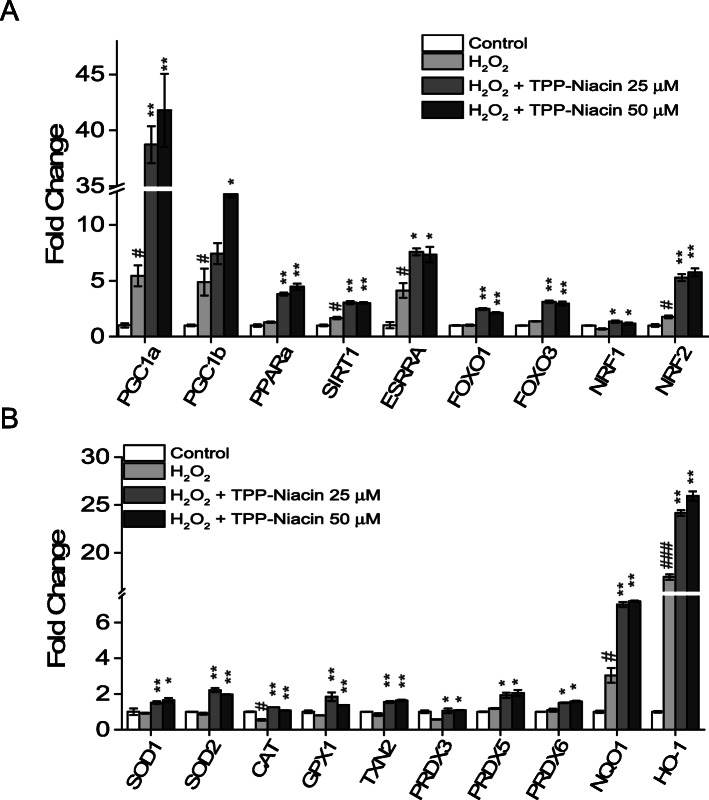
Involvement of PGC-1α/NRF2/NQO1/HO-1 axis for antioxidant effects of TPP-Niacin. Gene expression analysis by qPCR of transcription factors (**a**) and major antioxidant related genes (**b**). All gene expression data were analyzed using Student’s t-test. Statistical significance is represented as follows: # *P* < 0.05, ### *P* < 0.01 versus control group; ∗*P* < 0.05, ∗∗*P* < 0.01, and ∗∗∗*P* < 0.001 versus the H_2_O_2_-treated group

The transcriptional coactivator, peroxisome-proliferator-activated receptor-gamma coactivator 1α (PGC-1α) is a potent moderator of mitochondrial function, including oxidative phosphorylation and mitochondrial biogenesis, in RPE cells [24]. To further elucidate the involvement of PGC-1α in the protective effects of TPP-Niacin, the expression of PGC-1α related genes and antioxidant genes were assessed by RT-qPCR. The expressions of PGC-1α and PGC-1β were strongly upregulated after treatment with TPP-Niacin. Additionally, the gene expressions of estrogen-related receptor alpha (ESRRA), forkhead foxO1 and 3 (FOXO1, FOXO3), NRF2, PPARα, and Sirt1 were significantly increased by TPP-Niacin (Fig. 6a). Consistent with the results of ELISA (as shown in Fig. 3), the TPP-Niacin (25 and 50 μM) pretreatment increased the expressions of SOD1, SOD2, CAT, and GPX in ARPE-19 cells compared to the H_2_O_2_ treated cells. In addition, the NRF2 downstream target genes, NAD(P)H: quinone oxidoreductase 1 (NQO1), heme oxygenase-1 (HO-1), were also measured by RT-qPCR. As shown in Fig. 6b, the TPP-Niacin strongly upregulated the expressions of HO-1 and NQO-1 but not that of the NOX genes (data not shown) in the H_2_O_2_-treated ARPE-19 cells. These results suggest that the detoxified effects of TPP-Niacin may be attributed to its action as a ROS scavenger, by which it increases the expression level of the antioxidant enzyme, via reducing oxidative damage.

## Discussion

Oxidative stress in the retina plays a major role in the pathogenesis of dry AMD. While antioxidant defense systems in the retinal cells are appropriate under normal states, strong oxidative stress disintegrates the normal antioxidant systems and result in irreparable damage to the retina. It has been reported that the use of additional antioxidants reduces oxidative stress and preserves retinal function while avoiding oxidative damage [25, 26]. In addition, experimental and clinical studies suggest that consuming high doses of antioxidants, such as lutein, β-carotene, vitamins, and zinc supplements, possibly protect against and curtail the progression of AMD and vision loss [2]. In the present study, we demonstrated the improved protective effects of TPP-Niacin, a mitochondrial targeting compound, for the first time against oxidative damage in human RPE cells. At the mitochondrial level, the TPP-Niacin exerts improved protective effects via mediation of the MMP and its related effector genes, including OXPHOS, mitochondrial dynamics, and mitochondrial DNA replication and transcription. Notably, TPP-Niacin is capable of prevention against oxidative damage by incrementing the expression levels of antioxidant enzymes, mainly HO-1 and NQO-1, via upregulation of PGC-1α and NRF2 in the ARPE-19 cells. Furthermore, TPP-Niacin provides better protection than niacin against oxidative damage in ARPE-19 cells, therefore underscoring the potential use of TPP-Niacin as a possible therapeutic agent for AMD, a disease that initiated by cell death from oxidative stress and RPE dysfunction.

RPE cells are one of the types of cells that consume high amounts of energy, exist in the back of the photoreceptor cells, and have the most commonplace oxidative-damaged compositions in the retina. H_2_O_2_ is a critical factor in producing oxidative damage and cell deaths in various cell types, including retinal cells [27]. In the present study, H_2_O_2_ was used to test ARPE-19 cells to generate oxidative stress and cell cytotoxicity to imitate the onset of dry AMD. As noted in the viabilities and LDH assays, pretreatment with TPP-Niacin in the ARPE-19 cells significantly increased the cell viability against H_2_O_2_-induced cell death, whereas TPP-Niacin reduced cell death by oxidative damage. Intriguingly, TPP-Niacin treatment alone was able to slightly increase the growth of the ARPE-19 cells compared to the parent compound (Supplementary Figure 1a).

Intracellular accumulation of ROS is related with oxidative stress and dysfunction of RPE cells [28]. Reduction of intracellular ROS may protect the RPE cells from oxidative damage [5, 29]. The results of this study confirmed that TPP-Niacin markedly diminished H_2_O_2_-induced intracellular ROS levels in RPE cells, as observed via H_2_DCF-DA and DHE staining. Major antioxidant enzymes exist, including Cu/Zn-superoxide dismutase (cytosolic SOD, SOD1), manganese superoxide dismutase (mitochondrial SOD, SOD2), catalase, and glutathione peroxidase (GPx). The SOD converts superoxide to oxygen and hydrogen peroxide, whereas catalase and GPx transform hydrogen peroxide into H_2_O and O_2_ [28, 29]. The present study demonstrated that pre-incubation with TPP-Niacin increased SOD1 and SOD2 compared to the H_2_O_2_ group, thus suggesting that TPP-Niacin could combat oxidative stress. Additionally, the TPP-Niacin significantly increased catalase and GPx activities that were decreased by H_2_O_2_ in the ARPE-19 cells. These data indicate that TPP-Niacin may retain the ability to indirectly scavenge oxygen free radicals. Consequently, TPP-Niacin may reduce H_2_O_2_-induced oxidative stress in ARPE-19 cells by decreasing the intracellular ROS status and by eliminating oxygen free radicals. In addition, we observed that ARPE-19 cells pretreated with niacin and TPP-Niacin markedly reduced the H_2_O_2_-induced ROS production. As expected, the TPP-Niacin exerted a somewhat higher preventive effect against oxidative damage, as shown by a 10% increment in cell viability and 17% decrement in ROS level compared to niacin-treated cells, respectively. The antioxidant activities of TPP-Niacin are at a slightly higher level compared to the parent compound, suggesting that the mitochondria-targeting TPP-Niacin is an effective derivative of the parent compound.

The pathological changes of mitochondrial-related dysfunctions, including accumulation of ROS and superoxide in the mitochondria and MMP (△Ψm) reduction, were discovered in AMD [25]. In other mitochondrial targeting compounds [10, 14, 30], we observed that pretreatment with TPP-Niacin significantly enhanced the MMP and improved the mitochondrial ultrastructure in a phenotypic analysis by EM, compared to H_2_O_2_ alone. Based on these data, we next analyzed the expressions of mitochondria-related genes, such as OXPHOS subunits, mitochondrial dynamics, and mitochondrial DNA replication and transcription genes. Our results showed that TPP-Niacin significantly upregulated COX4I1, COX5B, NDUFB as well as MFN1, MFN2, TFAM, and POLG genes; thus, these mitochondrial specific effects of TPP-Niacin could lead to improved mitochondrial function and biogenesis against oxidative stress by H_2_O_2_.

Peroxisome proliferator-activated receptor gamma coactivator 1-alpha (PGC-1α) and -beta (PGC-1β) are transcriptional coactivators that control mitochondrial metabolism and functions in various tissues [31], including the retina [27, 32, 33]. To intermediate their functions, the PGC-1α isoforms cooperate with transcription factors, such as ESRRA, peroxisome proliferator-activated receptor α, γ (PPARα, γ), FOXO1, FOXO3, and nuclear respiratory factors 1 (NRF1) and Nfe212 (nuclear factor erythroid 2-related factor 2, NRF2) to control respiration, mitochondrial biogenesis, and expression of antioxidants [27, 34]. PGC-1α is necessary for the generation of ROS scavenging enzymes, including SOD1, SOD2, GPx, and CAT [35, 36]. Recently, several studies have shown that superoxide dismutase 2 (SOD2), an enzyme detoxifying the excessive accumulation of mitochondrial ROS, was turned on by PGC-1α in the RPE cells [27, 37]. Therefore, to determine the possible pathways of protective effects of the TPP-Niacin, we examined the gene expressions of PGC-1α related genes and observed that PGC-1α and PGC-1β were robustly upregulated by TPP-Niacin compared to the H_2_O_2_-induced oxidative damage group. In addition, when examining the potential downstream transcription factors responsible for these changes, ESRRA, FOXO1 and 3, and NRF1 and 2 were found to be upregulated by TPP-Niacin treatment.

On further investigating the possible mechanisms associated with the protective ability of TPP-Niacin, it appears that HO-1 and NQO-1, which are downstream targets of NRF2 signaling, play major roles in the prevention of oxidative damage in the cells [38, 39]. Recently, many studies have reported that the activation of NRF2/HO-1 signaling is required to alleviate oxidative damages in RPE cells [40–45]. In this study, it was speculated that the antioxidative effects of TPP-Niacin could be combined with PGC-1α and NRF2 signaling. The results of the present study show that TPP-Niacin protects the ARPE-19 cells from H_2_O_2_-induced oxidative damage by activating NRF2 signaling through upregulation of the expression of NRF2, NQO-1, and HO-1.

Initially, we thought that TPP-Niacin had an effect on the nanoconcentration state, as well as those of other mitochondrial targeting compounds, but TPP-Niacin showed antioxidant effects in the range of 10–200 μM. However, as shown in the comparison data with niacin, TPP-Niacin is more effective than its original chemical against oxidative damage in RPE cells. These results support TPP-Niacin as a potent antioxidant against oxidative stress compared to niacin and suggest that its improved protective effects are exerted via regulation of mitochondrial dynamics and antioxidant mechanisms. Contrary to expectations, the increment of antioxidant enzymes after induction of oxidative stress was in agreement with the study where the expression levels of HO-1 increased in ARPE-19 cells after H_2_O_2_ treatment [43, 44, 46–48]. However, some studies have presented that H_2_O_2_ treatment significantly reduces the expression levels of PGC-1α, HO-1, and NQO-1 in ARPE-19 cells [37, 40]. The variances between these studies may be owed to the use of other concentrations of stimuli as well as the treatment time, which can control cellular response. As in reported research, it is well established that niacin exerts significant antioxidant, anti-inflammatory and anti-apoptotic activities in a variety of cells and tissues [19, 20, 49–52]. Our study thus far has only been applied to focus upon the improved antioxidant effects of TPP-Niacin, in terms of mitochondrial and ROS regulation. Further data collection would be needed to determine exactly how TPP-Niacin affects with antioxidant effect via mitochondrial biogenesis and dynamics. Additionally, when we exam the TPP-Niacin’s own effects on normal ARPE-19 cells, as shown in Supplementary Figure 4, TPP-Niacin was not significantly changed of ARPE-19 cells compared with the control group in LDH, ROS, and MMP assay (Supplementary Figure 4 a-d). However, according to gene expression results, TPP-Niacin has significantly enhanced the SOD2 expression level assessed by RT-qPCR (Supplementary Figure 4e). These results indicated that TPP-Niacin mediated cytoprotective activities that could be linked to the mitochondrial function on not only the normal state but also the oxidative stress.

In conclusion, this study shows that TPP-Niacin is an improved protective antioxidant than niacin against oxidative damage to ARPE-19, cells via the reduction of ROS levels and protection against oxidative-stress-induced cell death. The signal mechanisms by which TPP-Niacin presented such effects involve regulation of the mitochondrial quality control and transcriptional factors such as PGC-1α and NRF2, as well as a boost in the antioxidant molecules. These results provide the first experimental evidence for TPP-Niacin as a possible therapeutic agent in the prevention of AMD. Further studies are needed to determine its physiological functions and biological efficacies in both primary human RPE cells (at least fully differentiated ARPE-19 cell models) and in vivo models, as well as target identification in the near future.

## Supplementary Information

**Additional file 1.** Comparison data between niacin and TPP-Niacin for this study can be found in the additional supplementary materials.

## Data Availability

The datasets used and analyzed during the current study are available from the corresponding author on reasonable request.

## Abbreviations

AMD: Age-related macular degeneration
RPE: Retinal pigment epithelium
TPP: Triphenylphosphonium
ROS: Reactive oxygen species
VEGF: Vascular endothelial growth factor
LDH: Lactate dehydrogenase
MMP: Mitochondrial membrane potential
NAC: N-acetyl-cysteine
CCCP: Carbonyl cyanide m-chlorophenyl hydrazone
H_2_DCF-DA: 2,7-dichlorofluorescein diacetate
DHE: Dihydroethidium
TMRE: Tetramethylrhodamine, ethyl ester
SOD1 and SOD2: Superoxide dismutase 1 and 2
CAT: Catalase
GPx: Glutathione peroxidase
EM: Electron microscopy
OXPHOS: Oxidative phosphorylation
ATP5O: ATP synthase subunit O
COX4I1: Cytochrome c oxidase subunit 4 isoform 1
COX5b: Cytochrome c oxidase subunit 5B
NDUFB5: NADH dehydrogenase (ubiquinone) 1 beta subcomplex, 5
FIS1: Fission 1
MFN1 and 2: Mitofusin 1 and 2
POLG: Mitochondrial DNA replication (polymerase (DNA directed), gamma
TFAM: Transcription factor a, mitochondrial
PGC-1α: Peroxisome-proliferator-activated receptor-gamma coactivator 1α
ESRRA: Estrogen-related receptor alpha
PPARα, γ: Peroxisome proliferator-activated receptor α, γ
FOXO1: Forkhead foxO1
FOXO3: Forkhead foxO3 and NRF1 nuclear respiratory factors 1
NRF2: Nfe212 (nuclear factor erythroid 2-related factor 2
HO-1: Heme oxygenase-1
NQO-1: NAD(P)H: quinone oxidoreductase 1
NOX: NADPH oxidase
