# Supplementary material for: Improved effect of a mitochondria-targeted antioxidant on hydrogen peroxide-induced oxidative stress in human retinal pigment epithelium cells
Source: BMC Pharmacol Toxicol. 2021 Jan 20;22:7. doi: 10.1186/s40360-020-00471-w (PMC7819191; doi:10.1186/s40360-020-00471-w)
Supplement: Supplementary file 1 — Additional file 1. Comparison data between niacin and TPP-Niacin for this study can be found in the additional supplementary materials. [file 40360_2020_471_MOESM1_ESM.pdf]

Improved Effect of a Mitochondria-Targeted Antioxidant on Hydrogen-Peroxide-Induced Oxidative Stress in Human Retinal Pigment Epithelial Cells

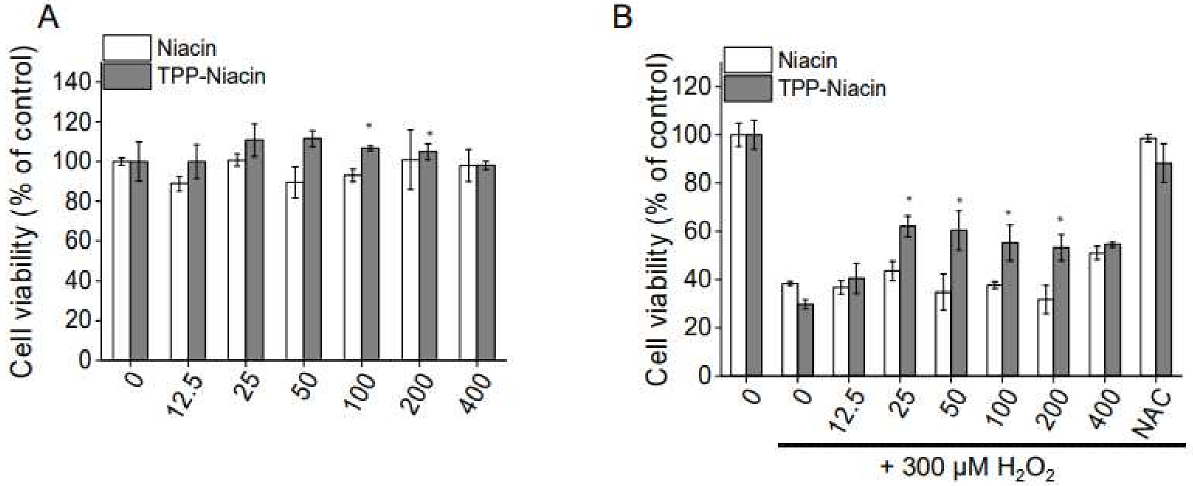

Supplementary Figure 1. Effects of niacin and TPP-Niacin on cell viabilities of ARPE-19 cells. (A) Different concentrations (range 12.5–400 μM) of niacin and TPP-Niacin were treated in ARPE-19 cells for 24 h without H_2O_2. (B) Cells were pretreated with niacin or TPP-Niacin for 2 h and then treated with H_2O_2 (300 μM) for 24 h, after which cell viabilities were evaluated by CCK-8. *$P < 0.05$ niacin versus the TPP-Niacin group were considered statistically significantly different.

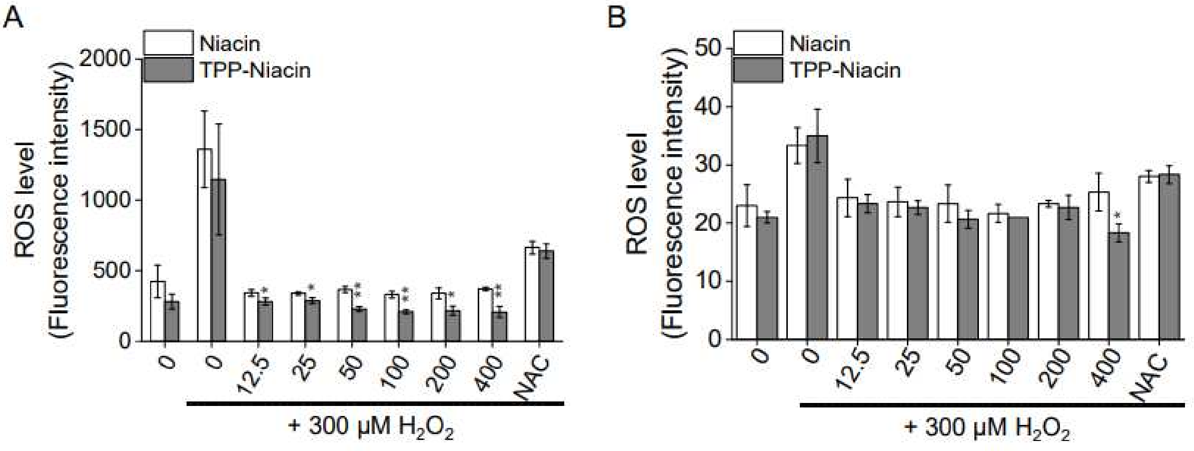

Supplementary Figure 2. Protective effects of niacin and TPP-Niacin against H_2O_2-induced ROS production in ARPE-19 cells. Cells were pretreated with various concentrations of niacin or TPP-Niacin for 24 h, followed by H_2O_2 treatment at 300 μM for 24 h. ROS generation was determined by H_2DCF-DA (A) and DHE (B) assays. *P < 0.05, **P < 0.01 niacin versus the TPP-Niacin group were considered statistically significantly different.

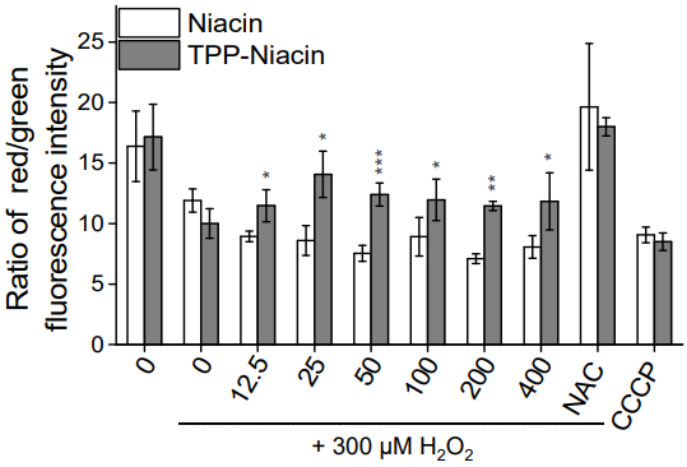

Supplementary Figure 3. TPP-Niacin improved mitochondrial membrane potential against H_2O_2-induced mitochondrial membrane potential (△Ψm) loss at various concentrations in ARPE-19 cells, but not niacin. ARPE-19 cells were treated with niacin or TPP-Niacin for 2 h, followed by a 300 μM H_2O_2 treatment for 24 h. MMP was analyzed by JC-10 assay. *P < 0.05, **P < 0.01, ***P < 0.001 niacin versus the TPP-niacin group were considered statistically significantly different.

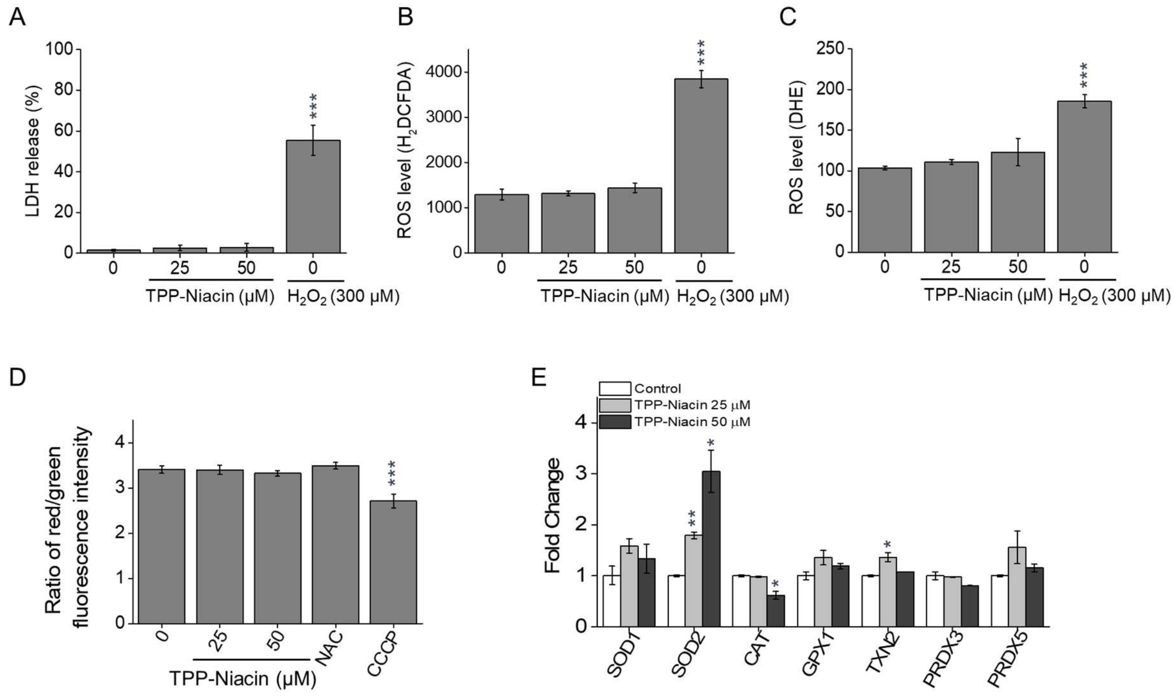

Supplementary Figure 4. Evaluation of TPP-Niacin on the normal state of ARPE-19 cells. The cells were treated with TPP-Niacin at 25 and 50 μM or 0.1% DMSO (vehicle control) for 24 h without H_2O_2 (300 μM) or with H_2O_2 only treated-group, cytotoxicity was measured by the LDH release (A). ROS generation and mitochondrial function were measured by H_2DCF-DA (B) and DHE (C), and MMP (D). Gene expression was analyzed by qPCR of major antioxidant related genes (E). All data were analyzed using Student's t-test. *P < 0.05, **P < 0.01, ***P < 0.001 versus control group were considered statistically significant differences.
